# A predictive score for progression of COVID-19 in hospitalized persons: a cohort study

**DOI:** 10.1038/s41533-021-00244-w

**Published:** 2021-06-03

**Authors:** Jingbo Xu, Weida Wang, Honghui Ye, Wenzheng Pang, Pengfei Pang, Meiwen Tang, Feng Xie, Zhitao Li, Bixiang Li, Anqi Liang, Juan Zhuang, Jing Yang, Chunyu Zhang, Jiangnan Ren, Lin Tian, Zhonghe Li, Jinyu Xia, Robert P. Gale, Hong Shan, Yang Liang

**Affiliations:** 1 Department of Hematology, The Fifth Affiliated Hospital, Sun Yat-sen University, Zhuhai, Guangdong, China; 2 Department of Hematologic Oncology, Sun Yat-sen University Cancer Center, State Key Laboratory of Oncology in South China, Collaborative Innovation Center for Cancer Medicine, Guangzhou, Guangdong, China; 3 Department of Interventional Therapy, The Fifth Affiliated Hospital, Sun Yat-sen University, Zhuhai, Guangdong, China; 4 Department of Rheumatology, The Fifth Affiliated Hospital, Sun Yat-sen University, Zhuhai, Guangdong, China; 5 Department of Gastroenterology, The Fifth Affiliated Hospital, Sun Yat-sen University, Zhuhai, Guangdong, China; 6 Department of Pharmacy, The Fifth Affiliated Hospital, Sun Yat-sen University, Zhuhai, Guangdong, China; 7 Department of Nephrology, The Fifth Affiliated Hospital, Sun Yat-sen University, Zhuhai, Guangdong, China; 8 Department of Infectious Diseases, The Fifth Affiliated Hospital, Sun Yat-sen University, Zhuhai, Guangdong, China; 9 Haematology Research Centre, Department of Immunology and Inflammation, Imperial College London, London, UK

**Keywords:** Diagnosis, SARS-CoV-2

## Abstract

Accurate prediction of the risk of progression of coronavirus disease (COVID-19) is needed at the time of hospitalization. Logistic regression analyses are used to interrogate clinical and laboratory co-variates from every hospital admission from an area of 2 million people with sporadic cases. From a total of 98 subjects, 3 were severe COVID-19 on admission. From the remaining subjects, 24 developed severe/critical symptoms. The predictive model includes four co-variates: age (>60 years; odds ratio [OR] = 12 [2.3, 62]); blood oxygen saturation (<97%; OR = 10.4 [2.04, 53]); C-reactive protein (>5.75 mg/L; OR = 9.3 [1.5, 58]); and prothrombin time (>12.3 s; OR = 6.7 [1.1, 41]). Cutoff value is two factors, and the sensitivity and specificity are 96% and 78% respectively. The area under the receiver-operator characteristic curve is 0.937. This model is suitable in predicting which unselected newly hospitalized persons are at-risk to develop severe/critical COVID-19.

## Introduction

Approximately 10 percent of persons with SARS-CoV-2-infection are hospitalized because they develop severe/critical coronavirus disease (COVID-19)^1–3^. According to the interim guidance of the Centers for Disease Control and Prevention (CDC)^5^, in areas with sustained community-level outbreaks^4^, typically urban, most hospital admissions are for persons with severe/critical and/or comorbidities. This selection bias reflects limited acute care resources but is not representative of the global epidemiology of either SARS-CoV-2-infection or of COVID-19. Consequently, most prognostic and predictive scores using admission co-variates are for death from COVID-19 in persons with severe/critical COVID-19 rather than disease progression in persons with less severe disease^6,7^. For example, in a report from Italy of 1591 subjects, it was mentioned that all subjects were admitted to an intensive care unit (ICU), 99 percent of evaluable subjects required respiratory support, and the case fatality rate for ICU subjects was 26 percent^8^.

Understandably, most reports of large series of persons with COVID-19 are from urban centers where SARS-CoV-2-infection is an epidemic. However, as the numbers of confirmed cases and endemic areas increase every day, it is with high to certain probability that more and more areas currently with sporadic or clustered cases will eventually become areas with sustained community-level outbreaks and would involve hospitalizations for less severe disease under current containments. This is what we have seen in the historical influenza epidemics according to CDC’s pandemic interval framework^9^. Under these circumstances, a precise and convenient triage strategy would be especially important in allocating health care capacity. To address this issue, we studied outcomes of 98 consecutive subjects with COVID-19 in a region of 2 million persons where most admissions were for persons with mild or moderate COVID-19. We were able to use these data to develop a predictive model of the risk of progression to severe/critical COVID-19. These data may help physicians prioritize use of medical resources accordingly.

## Results

From January 17 to February 13, 2020, 98 patients with COVID-19 in Zhuhai were admitted to the hospital (Table 1); 46 subjects were male. Their median age was 47 years (interquartile range [IQR], 34–62; range, 10 months to 80 years). From the total number of patients, 77 had traveled to an epidemic area and 18 had contact with a SARS-CoV-2-infected person; 45 subjects had comorbidities on admission, including hypertension (*N* = 17), diabetes (*N* = 7), cancer (*N* = 5), tuberculosis (*N* = 2), and chronic kidney disease (*N* = 2). The median duration from symptoms onset to admission was 3 days (IQR, 1.0–5.3). On admission, 13 subjects were classified as having mild disease, 79 were classified as moderate, and 3 were classified as patients with severe COVID-19. None of these subjects were critical. (3 subjects were not classified on admission).

**Table 1:** Baseline characteristics of patients with COVID-19 on admission by severity at triage and severity during hospitalization.

Parameters		No. (%)							
	Entire cohort^a^ (*N* = 98)		Severity at triage				Severity during hospitalization		
		Mild (*n* = 13)	Moderate (*n* = 79)	Severe (*n* = 3)	N/A (*n* = 3)	*P*-value	Mild/Moderate (*n* = 74)	Severe/Critical (*n* = 24)	*P*-value
Age, years (median, IQR)	46.5 (34.3–62.0)	34.0 (29.0–42.5)	50.5 (36.0–60.5)	78.0 (65.0–80.0)	37.0 (6.0–63.0)	0.004	39.0 (31.3–56.3)	63.0 (46.0–66.5)	<0.001
Sex						0.55			0.06
Female	53 (54.1)	5 (38.5)	45 (57.0)	1 (33.3)	2 (66.7)		44 (59.5)	9 (37.5)	
Male	45 (45.9)	8 (61.5)	34 (43.0)	2 (66.7)	1 (33.3)		30 (30.5)	15 (62.5)	
Epidemiological history	(*N* = 92)		*n* = 74	*n* = 2		0.04	*n* = 73	*n* = 19	0.06
Travel to epidemic area before onset	71 (77.2)	7 (53.8)	60 (81.1)	1 (50.0)	3 (100)		59 (80.8)	12 (63.2)	
Known sick contacts	17 (18.5)	6 (46.2)	11 (14.9)	1 (50.0)	0 (0)		14 (19.2)	3 (15.8)	
Unknown exposure	4 (4.3)	0 (0)	3 (4.1)	0 (0)	0 (0)		0(0)	4 (21.1)	
Comorbidities	44 (44.9)	3 (23.1)	37 (46.8)	2 (66.7)	2 (66.7)	0.27	30 (40.5)	14 (58.3)	0.13
Hypertension	17 (17.3)	1 (7.7)	15 (19.0)	1 (33.3)	0 (0)	0.54	9 (12.2)	8 (33.3)	0.05
Diabetes Mellitus	7 (7.1)	0 (0)	6 (7.6)	1 (33.3)	0 (0)	0.28	2 (2.7)	5 (20.8)	0.05
Malignancies	5 (5.1)	0 (0)	3 (3.8)	1 (33.3)	1 (33.3)	0.03	4 (5.4)	1 (4.2)	0.83
Tuberculosis	2 (2.0)	0 (0)	2 (2.5)	0 (0)	0 (0)	1.00	0 (0)	2 (8.3)	0.16
Chronic kidney diseases	2 (2.0)	1 (7.7)	1 (1.3)	0 (0)	0 (0)	0.35	1 (1.4)	1 (4.2)	0.42
Stroke	1 (1.0)	0 (0)	1 (1.3)	0 (0)	0 (0)	1.00	1 (1.4)	0 (0)	0.57
Coronary heart disease	1 (1.0)	0 (0)	1 (1.3)	0 (0)	0 (0)	1.00	1 (1.4)	0 (0)	0.57
Symptoms									
Paucisymptomatic	15 (15.3)	1 (7.7)	12 (15.2)	0 (0)	2 (66.7)	0.13	14 (18.9)	1 (4.2)	0.02
Fever	58 (59.2)	9 (69.2)	48 (60.8)	1 (33.3)	0 (0)	0.13	39 (52.7)	19 (79.2)	0.01
*T*_max_ on admission (°C, IQR)	38.0 (37.5–38.3)	38.0 (37.7–38.8)	38.0 (37.5–38.3)	37.6	N/A	0.36	37.8 (37.5–38.3)	38.0 (37.8–38.3)	0.50
Cough	17 (17.3)	2 (15.4)	14 (17.7)	1 (33.3)	0 (0)	0.83	14 (18.9)	3 (12.5)	0.48
Sore throat	5 (5.1)	0 (0)	5 (6.3)	0 (0)	0 (0)	1.00	5 (6.8)	0 (0)	0.02
Diarrhea	4 (4.1)	0 (0)	2 (2.5)	1 (33.3)	1 (33.3)	0.04	2 (2.7)	2 (8.3)	0.36
SpO_2_ on admission, %, IQR (*N* = 97)^b^	98.0 (96.9–98.8)	98.0 (97.1–98.8)	98.2 (96.9–98.8)	94.0 (91.0–94.5)	98.0 (98.0–99.9)	0.04	98.3 (97.7–98.9)	96.1 (94.6–97.9)	0.03
Days to admission, IQR^c^	3.0 (1.0–5.3)	2 (1–5)	3 (1–5)	6 (5–12)	N/A	0.43	3 (1–6)	3 (1–4)	0.58

*COVID-19* coronavirus disease 2019, *IQR* interquartile range, *N/A* not available, *SpO*_*2*_ saturation of peripheral oxygen, *T*_*max*_ peak temperature.

^a^Three patients did not have severity stratification at triage.

^b^Missing data in 1 patient in Moderate group.

^c^Duration from symptom onset to admission in symptomatic patients, from probable exposure to admission in asymptomatic patients.

During hospitalization, four subjects received mechanical ventilation; ten subjects in the moderate severity cohort were administered corticosteroids. Further, 17 subjects (15 in the moderate cohort) were administered chloroquine, 12 (11 in the moderate cohort) subjects were administered lopinavir/ritonavir (LPV/r), and 13 subjects were administered intravenous immunoglobulin.

At the time of final follow-up (median 55 days from admission; IQR, 52–58; range, 37–79 days), the highest COVID-19 severity scores were mild in 8 subjects, moderate in 66, severe in 19, and critical in 5. Severity grade shifts are summarized in Table 2. Among 92 mild and moderate patients on admission, 21 (22.8%) progressed during hospitalization, including 3 with critical illness. The progression rates were 15.4% and 24.1% in the mild and moderate groups respectively. The median duration of hospitalization or interval to death was 18 days, which was not significantly different among the severity cohorts. The median duration of virus shedding was 8 days (IQR: 4–10 days; range, 1–19 days), and was similar among the severity cohorts. Detailed laboratory results of patients with COVID-19 on admission by severity is illustrated in Table 3.

**Table 2:** Overview of therapeutic interventions and clinical outcomes for patients with COVID-19 by severity at triage.

	No. (%)					
	Entire cohort (*n* = 98)	Severity at triage				
		Mild (*n* = 13)	Moderate (*n* = 79)	Severe (*n* = 3)	N/A (*n* = 3)	*P*-value
Therapeutic approach						
Mechanical ventilation	4 (4.1)	0 (0)	2 (2.5)	2 (66.7)	0 (0)	0.01
Glucocorticoid	10 (10.2)	0 (0)	10 (12.7)	0 (0)	0 (0)	0.66
Chloroquine	17 (17.3)	1 (7.7)	15 (19.0)	0 (0)	1 (33.3)	0.54
LPV/r	12 (12.2)	1 (7.7)	11 (13.9)	0 (0)	0 (0)	1.00
IVIG	13 (13.3)	2 (15.4)	9 (11.4)	1 (33.3)	1 (33.3)	0.18
Final severity stratification during hospitalization						<0.001
Mild	8 (8.2)	6 (46.2)	1 (1.3)	0 (0)	1 (33.3)	
Moderate	66 (67.3)	5 (38.5)	59 (74.7)	0 (0)	2 (66.7)	
Severe	19 (19.4)	2 (15.4)	16 (20.3)	1 (33.3)	0 (0)	
Critical	5 (5.1)	0 (0)	3 (3.8)	2 (66.7)	0 (0)	
Outcomes						
Length of stay in hospital, days, IQR	18.0 (14.8–25.3)	20.0 (16.0–23.5)	18.0 (15.0–26.0)	19.0 (9.0–23.0)	13.0 (11.0–19.0)	0.54
Died in hospital	1 (1.0)	0 (0)	0 (0)	1 (33.0)	0 (0)	0.06
Discharged from hospital	96 (98.0)	13 (100)	78 (98.7)	2 (66.7)	3 (100)	0.16
Days to negativity, IQR^a^	8.0 (4.0-10.0)	9 (3–12)	8 (5–10)	4 (4–9)	N/A	0.64

*COVID-19* coronavirus disease 2019, *N/A* not available, *LPV/r* lopinavir/ritonavir, *IVIG* intravenous immunoglobulin, *IQR* interquartile range.

^a^Duration from admission to the date that patients tested a second PCR negativity in throat or nasal swab specimen.

**Table 3:** Laboratory results of patients with COVID-19 on admission by severity during hospitalization.

Parameters (median, IQR)	Entire cohort (*n* = 98)	Severity during hospitalization		
		Mild/Moderate (*n* = 74)	Severe/Critical (*n* = 24)	*P*-value
Hematologic				
White-cell count (x10^9^/L)	5.0 (4.0–6.5)	5.0 (4.2–6.4)	5.0 (3.6–6.6)	0.71
Lymphocyte count (x10^9^/L)	1.6 (1.1–2.1)	1.7 (1.3–2.2)	1.1 (0.6–1.5)	0.001
Hemoglobin (g/L)	136.5 (125.8–148.0)	137.0 (126.8–146.0)	135.0 (119.3–149.8)	0.42
Platelet (x10^9^/L)	194.5 (164.5–251.0)	209.5 (178.0–272.0)	167 (133.5–197.5)	<0.001
CD4 + T cell count (/mm^3^)	547 (404–751)	597 (467–841)	432 (220–547)	0.004
CD8 + T cell count (/mm^3^)	337 (236–492)	402.5 (293–524.8)	216 (122–289)	<0.001
Liver function				
Alanine aminotransferase (U/L)	15.5 (11.1–28.0)	15.0 (10.0–25.8)	21.7 (13.8–42.1)	0.05
Aspartate aminotransferase (U/L)	20.4 (15.3–29.8)	19.0 (14.4–25.3)	27.3 (18.5–38.9)	0.01
Albumin (g/L)	38.7 (36.0–41.5)	39.0 (36.3–42.3)	36.7 (35.5–41.1)	0.53
Coagulation				
Prothrombin time (s)	12.0 (11.5–12.6)	11.8 (11.4–12.5)	12.5 (12.0–13.1)	0.01
International normalized ratio	1.05 (1.01–1.11)	1.04 (1.00–1.10)	1.09 (1.05–1.14)	0.03
Activated partial thromboplastin time (s)	31.6 (29.1–33.5)	31.6 (28.8–33.1)	32.1 (29.7–34.2)	0.81
Fibrinogen (g/L)	3.03 (2.56–3.72)	2.87 (2.51–3.41)	3.77 (2.88–4.29)	0.001
Thrombin time (s)	13.8 (13.0–15.1)	14.2 (13.3–15.1)	13.3 (12.5–14.1)	0.35
Blood chemistry				
Sodium (mmol/L)	140.0 (138.0–142.0)	139.0 (137.0–141.5)	142.0 (139.3–143.0)	0.02
Blood glucose (mmol/L)	5.34 (4.90–6.48)	5.34 (4.90–6.79)	5.36 (4.89–6.16)	0.69
Kidney function				
Blood urea nitrogen (μmol/L)	3.70 (2.90–4.30)	3.50 (2.70–4.20)	3.90 (3.65–4.55)	0.30
Creatine (mmol/L)	59.6 (47.8–71.6)	57.0 (47.0–70.8)	67.1 (53.2–75.2)	0.84
Inflammatory				
Lactate dehydrogenase (U/L)	164.0 (142.5–201.5)	158.0 (135.0–196.0)	180.5 (156.0–215.8)	0.02
Hydroxybutyric dehydrogenase (U/L)	129.0 (108.0–155.0)	128.0 (107.0–155.0)	141.5 (119.3–175.8)	0.06
Lactate (mmol/L)	1.50 (1.10–1.95)	1.50 (1.20–1.90)	1.05 (0.80–2.20)	0.61
C-reactive protein (mg/L)	4.10 (0.56–17.60)	1.16 (0.35–5.60)	26.70 (12.35–44.25)	0.002

*COVID-19* coronavirus disease 2019, *IQR* interquartile range.

**Fig. 1:**
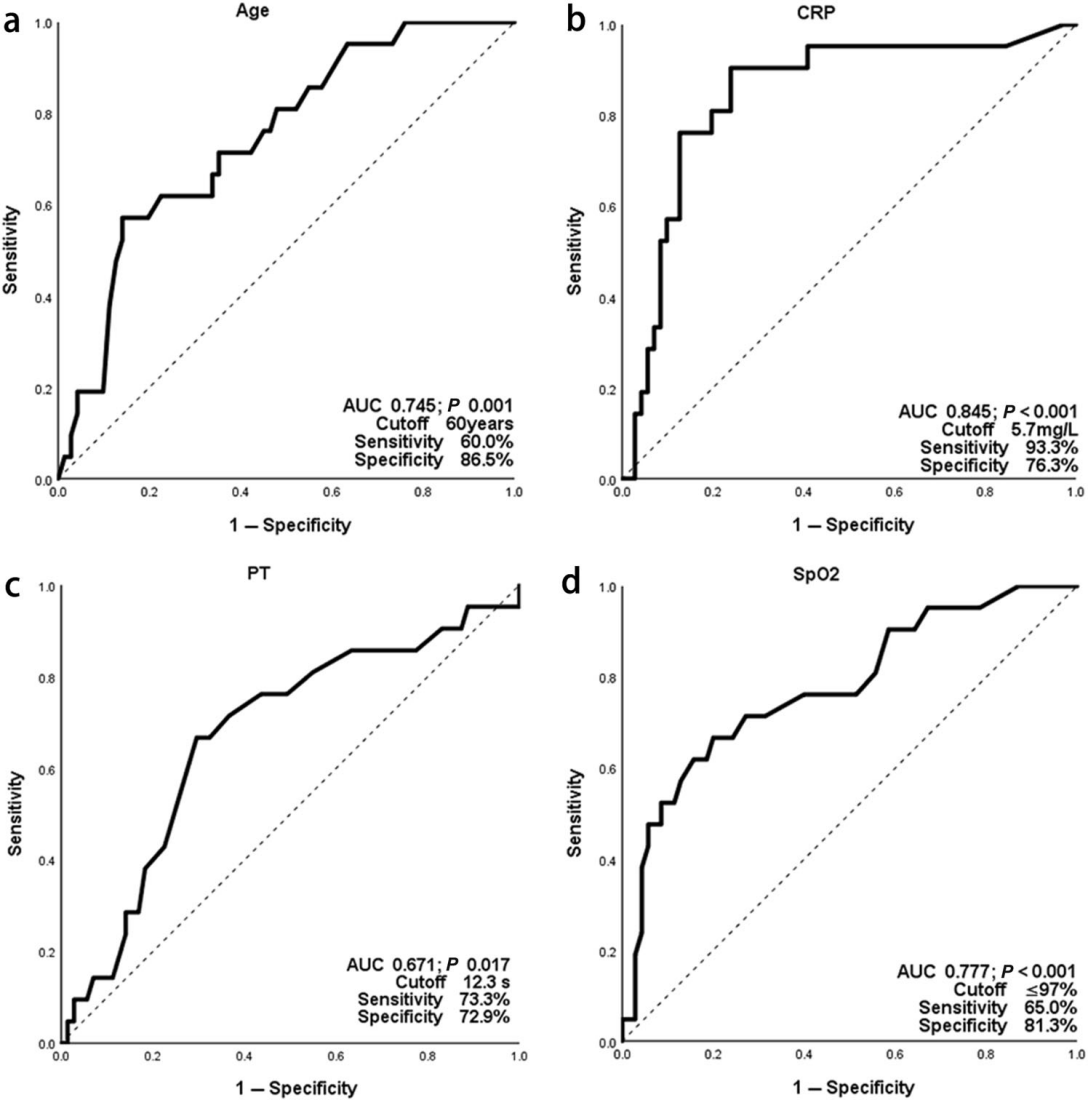
Receiver operating characteristic curves to identify cut-offs of co-variates on admission in patients with mild/moderate SARS-CoV-2-infection. These four co-variates were entering the final model and shown here, other co-variates were shown in Supplementary Fig. 1. **a** Age; **b** CRP; **c** PT; **d** SpO_2_. CRP c-reactive protein, PT prothrombin time, SpO_2_ blood oxygen saturation.

**Fig. 2:**
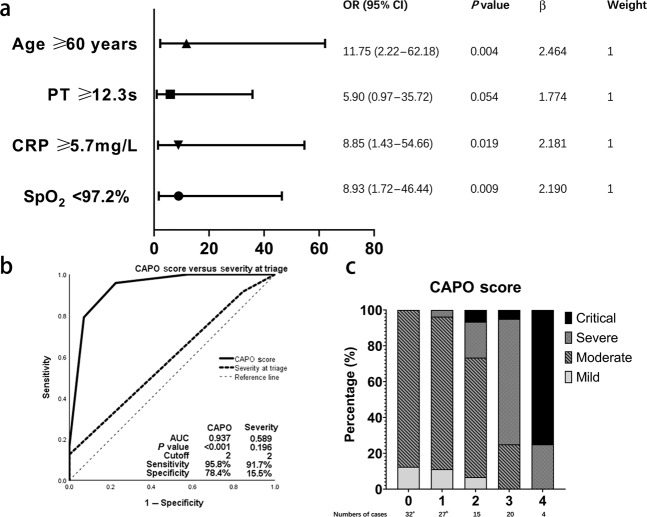
Development and performance of predicting score of progression risk in patients with mild/moderate SARS-CoV-2-infection. **a** Multivariate analysis associated with progression to severe/critical illness. **b** Characteristic curves of predicting score and severity at triage for prediction of progression. **c** Distributions of final severity stratification by predicting score on admission. CRP c-reactive protein, PT prothrombin time, SpO_2_ blood oxygen saturation, OR odds ratio, CAPO CRP, Age, PT, SpO_2_.

In subjects with mild or moderate severity disease on admission (*N* = 95), we interrogated co-variates associated with risk of progression to severe/critical disease. Some binary co-variates were excluded because of low sensitivity and specificity. Continuous variables were tested in receiver-operator characteristic (ROC) curves to identify cutoff values (Fig. 1 and Supplementary Fig. 1), transformed into categorical variables, and entered in multivariate backward stepwise logistic regression analysis with clinical co-variates significantly associated with the risk of progression (as shown in Tables 1 and 3). Several duplicates and co-linear covariates were excluded such as International Normalized Ratio, CD4 and CD8-positive cell concentrations. C-reactive protein (CRP) > 5.75 mg/L, prothrombin time (PT) > 12.3 s, age > 60 years, and blood oxygen saturation (SpO_2_) < 97% correlated with a severer disease characteristic, showing a clinical value in categorizing patients with a higher risk of progression to severe/critical diseases. Co-variates, odds ratios and 95% confidence intervals are shown in Fig. 2. The score of each point was defined as the relative weights assigned according to the regression coefficient of each categorical co-variate, namely 1 point for each. The area under the ROC curve (AUROC) of the score was 0.937. The cutoff value in dividing subjects into high- and low-risk groups with the potential risk for progressing to severe/critical cases was 2, with a sensitivity of 96% and specificity of 78%. The hazard ratio of progression to severe/critical COVID-19 in subjects with a score ≥2 was 42 (11–164) compared with subjects whose score was <2. 59 percent (43, 73%) subjects with a score ≥2 developed severe/critical COVID-19 compared with 2 percent (0.3, 9.0%, *P* < 0.001) of subjects with a score <2.

## Discussion

In our study of 98 consecutive, unselected subjects with SARS-CoV-2-infection and COVID-19 (including all hospitalized persons) in an area of 2 million people with sporadic or clustered cases, we identified four admission co-variates that were significantly associated with progression to severe/critical disease. We used these co-variates to develop a predictive score that identified subjects with a 40-fold increased risk of progression of COVID-19 to a severe/critical stage with a sensitivity of 96% and a specificity of 78%. The AUROC was 0.937. Our model is simple to use based on readily available co-variates.

There are several reports of prognostic and predictive scores of outcomes of COVID-19, although most studies have important biases and are not representative of real-world experience with the SARS-CoV-2 pandemic and with COVID-19^10^. For example, in regions where authorities imposed home isolation and social distancing, most persons with mild/moderate COVID-19 were not hospitalized^11^. On the other hand, in urban regions with large numbers of cases of COVID-19 and limited intensive care resources such as mechanical ventilation, most hospital admissions were for persons with severe/critical COVID-19 alone^12–14^. There were also obvious selection biases as to why and where people were hospitalized in these studies, and hospitalized persons from a center or a few centers were unlikely representatives of the distribution of cases of COVID-19 in a region, especially a region with sporadic or clustered cases of SARS-CoV-2-infection^15,16^. In several studies, there was censoring of subjects still in-hospital, which biased the interpretations of results accordingly^12,17^. In contrast, we were able to identify every patient of COVID-19 in our area, all of whom were actually hospitalized. We also conducted a complete follow-up of all the subjects. These biases are obvious when we consider the 1 death in our study versus an average of 10–20 percent in other studies^14,18^. Our subjects were more likely to be similar to a typical non-epidemic setting of exposure to SARS-CoV-2; therefore, our prognostic score is more likely to be widely useful^1,19,20^.

Similar to other studies, we found that age, CRP, and SpO_2_ on admission correlated with outcomes^12,21–23^. However, our study differed from other predicting tools^12,17,21,22^, wherein we identified a new risk factor, PT. Previous studies indicate that coagulation disorders are common in patients with severe COVID-19^24,25^ and are associated with an increased risk of acute respiratory distress syndrome^26^. We suggested early monitoring of PT to predict the likelihood of progression.

In conclusion, our study had limitations including its retrospective design, relatively few subjects, and no validation cohort. We also lacked detailed data on post-admission interventions. However, other than oxygen supplementation and mechanical ventilation, none of the other interventions proved effective^27^. Also, our aim was to predict outcomes from admission to better allocate medical resources. Our score is easily implemented and should assist physicians to identify persons with COVID-19 on admission at the greatest risk to develop severe/critical disease.

## Methods

### Subjects

This retrospective observational study was conducted at the Fifth Affiliated Hospital of Sun Yat-sen University, the largest tertiary academic hospital in Zhuhai. The Institutional Review Board of the Fifth Affiliated Hospital of Sun Yat-sen University approved the study protocol and classified the study minimal-risk and voided requirement for informed consent. COVID-19 containment (including active detection and quarantine of close contacts and travelers from epidemic areas [Hubei province]) was led by the Chinese government^28^. All consecutive subjects with confirmed SARS-CoV-2-infection, determined by quantitative reverse transcription polymerase chain reaction (qRT-PCR; Novel Coronavirus [2019-nCoV][Real Time Multiplex RT-PCR Kit [Z-RR-0479-02-25, Shanghai ZJ Liferiver Bio-tech Co., Ltd. Shanghai, China]), between 17 January and 13 February 2020 were enrolled in the study. Subjects received therapy in accordance with the interim guidelines of the National Health Commission, China^29^. Lopinavir/ritonavir, chloroquine, and arbidol were administered to some subjects. Data lock was 21 March, 2020.

### Data

Demographic data, clinical symptoms, and laboratory results were collected and extracted from the hospital electronic medical records. Two investigators independently coordinated and integrated the data with discordances adjudicated by reviewing original records. Subject identifiers were deleted, thereby creating an anonymized dataset. Laboratory assessments included complete blood count with differential, liver and kidney function tests, coagulation tests, and C-reactive protein and lymphocyte subsets.

### Definitions

Criteria for the diagnosis of COVID-19 followed the interim guidelines of the National Health Commission, China^29^. A confirmed case was based on the exposure history, which included exposure to suspected cluster outbreaks, clinical manifestations (fever and/or respiratory symptoms), chest computed tomography imaging, and results of qRT-PCR for SARS-CoV-2 and anti-SARS-CoV-2 IgM and IgG antibodies using enzyme-linked immunosorbent assay. Classification of COVID-19 severity was based on the interim or 7th edition guidelines of the National Health Commission^29^. Severity was stratified on admission and revised based on disease progression during hospitalization. Outcomes were evaluated at the date of last follow-up, discharge or death, and by whether the subject required continued hospitalization. The highest severity during hospitalization was designed as the primary outcome to develop the predictive model for the likelihood of progression.

### Statistics

Descriptive statistics were used to summarize demographic data. Results were reported as medians and IQRs, means with standard deviations, or counts and frequencies Continuous variables were compared using the *t*-test or one-way analysis of variance (ANOVA). Categorical dependent parameters were compared using the chi-square test and Fisher’s exact test. Cut-off values were identified following Youden index of ROC curve. All tests were two-sided, and a *P-*value < 0.05 was considered significant. The Statistical Package for Social Sciences (SPSS) 25.0 software (*IBM*, Armonk, NY, USA), VassarStats (Vassarstats.net), and the GraphPad Prism (version 8.2.1) (*GraphPad Software*, San Diego, CA, USA) were used for statistical analyses and illustrations.

### Reporting summary

Further information on research design is available in the Nature Research Reporting Summary linked to this article.

## Supplementary information

Supplementary Information

Reporting Summary

## Data Availability

The data that support the findings of this study are available from the corresponding author upon reasonable request.
