# Supplementary material for: A predictive score for progression of COVID-19 in hospitalized persons: a cohort study
Source: NPJ Prim Care Respir Med. 2021 Jun 3;31:33. doi: 10.1038/s41533-021-00244-w (PMC8175565; doi:10.1038/s41533-021-00244-w)
Supplement: Supplementary file 1 — Supplementary Information [file 41533_2021_244_MOESM1_ESM.pdf]

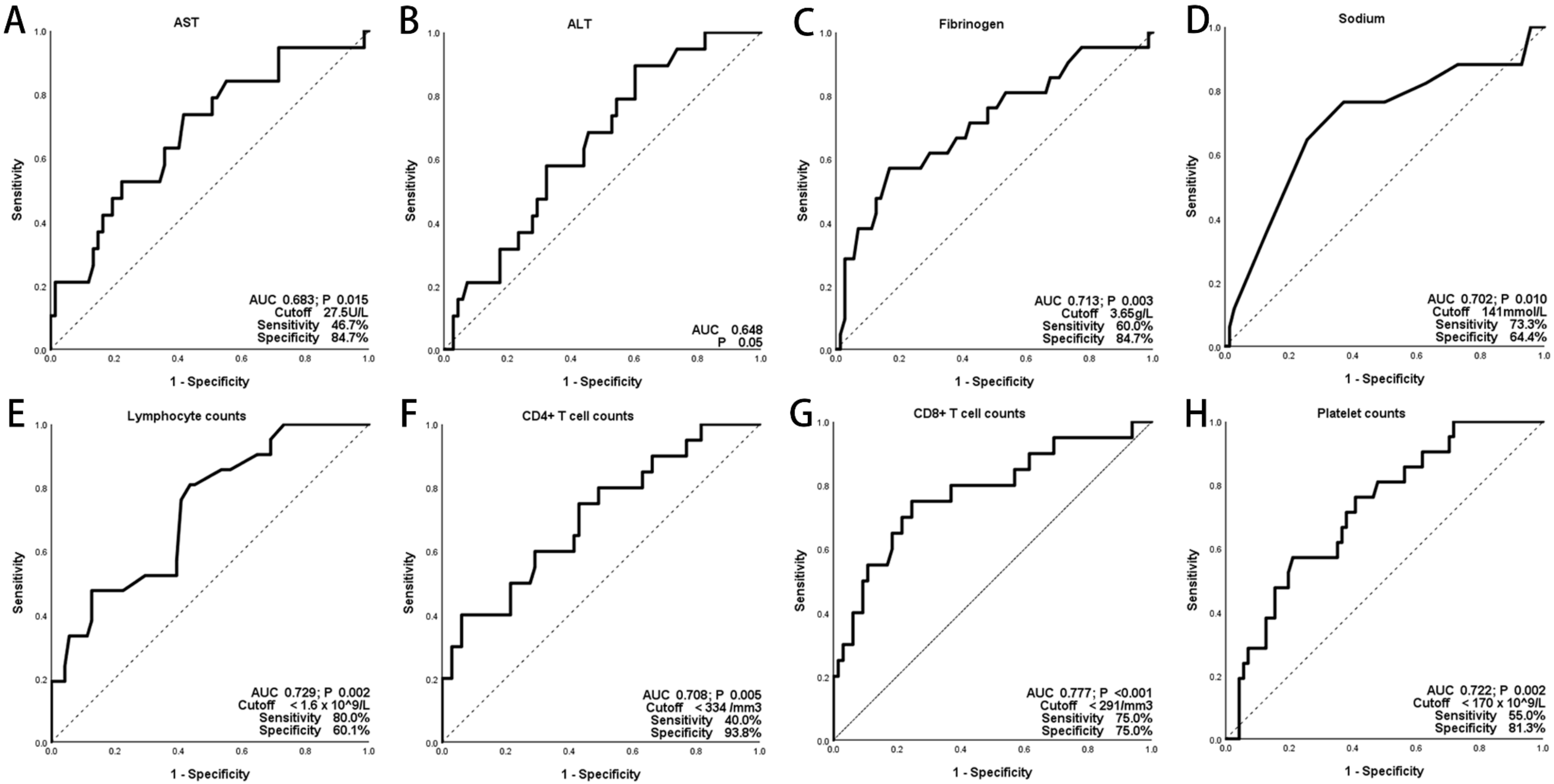

Supplementary Figure 1. Receiver Operating Characteristic curves to identify cut-offs of co-variates on admission in patients with mild/moderate SARS-CoV-2 infection. Co-variates that entering the final model were shown in Figure 1, others were shown here. A: AST; B: ALT; C: Fibrinogen; D: Sodium; E: Lymphocyte counts; F: CD4+ T cell counts; G: CD8+ T cell counts; H: Platelet counts. AST: aspartate aminotransferase; ALT: alanine aminotransferase.
